# Supplementary material for: Antileishmanial Activity and In Silico Molecular Docking Studies of Malachra alceifolia Jacq. Fractions against Leishmania mexicana Amastigotes
Source: Trop Med Infect Dis. 2023 Feb 14;8(2):115. doi: 10.3390/tropicalmed8020115 (PMC9960462; doi:10.3390/tropicalmed8020115)
Supplement: Supplementary file 1 [file tropicalmed-08-00115-s001.zip › tropicalmed-2168795-supplementary.pdf]

Antileishmanial activity and in silico molecular docking studies of *Malachra alceifolia* Jacq. Fractions against *Leishmania mexicana* amastigotes

Leonor Cervantes-Ceballos[1]*§, Jairo Mercado-Camargo[1]§, Esther del Olmo-Fernández [2], María Luisa Serrano-García[3], Sara M. Robledo[4] and Harold Gómez-Estrada[1].

[1]Grupo de Investigación en Química Orgánica Medicinal. Facultad de Ciencias Farmacéuticas, Universidad de Cartagena, Campus de Zaragocilla, 130001, Cartagena-Colombia.
[2]Departamento de Ciencias Farmacéuticas, Área de Química Farmacéutica, Facultad de Farmacia, Centro de Enfermedades Tropicales de la Universidad de Salamanca (CIETUS), Instituto de Investigación Biomédica de Salamanca (IBSAL), Universidad de Salamanca, Campus Miguel de Unamuno, 37007 Salamanca, Spain.
[3]Unidad de Química Medicinal, Facultad de Farmacia, Universidad Central de Venezuela, Caracas, Venezuela.
[4]Programa de Estudio y Control de Enfermedades Tropicales PECET. Facultad de Medicina, Universidad de Antioquia. Medellín-Colombia.

*Correspondence: lcervantesc@unicartagena.edu.co
§ These authors contributed equally to this work.

Table S1. Qualitative Phytochemical screening of *M. alceifolia* Jacq., extract and fractions

**Extract/ fractions**	Alc			Gc			Cum	Flv		Tan		**Ter/Est**		Sap		Ant
	D	M	W	K	R	KK	B	Sh	Ci	Fe	Gs	LB	SW	Es	VS	BO
**MA-IEt**	-	-	-	-	-	-	+	+	+	+	+	**+**	**+**	-	-	-
**MA-IH**	-	-	-	-	-	-	+	-	-	+	+	**+**	**+**	-	-	-
**MA-IC**	-	-	-	+	+	+	+	+	+	+	+	**+**	**+**	-	-	-
**MA-IA**	-	-	-	-	-	-	+	+	+	+	+	**+**	**+**	-	-	-
**MA-IM**	-	-	-	-	-	-	+	+	+	+	+	**+**	**+**	-	-	-

Total ethanolic extract (MA-IEt); *n*-hexane fraction (MA-IH); chloroform fraction (MA-IC); ethyl acetate fraction (MA-IA) and Methanol fraction (MA-IM). Alc: Alkaloids, Gc: Cardiac Glycosides, Cum: Coumarins, Flv: Flavonoids, Tan: Tanins, Ter/est: Terpenoids/ Sterols, Sap: Saponins, Ant: Anthraquinones. D: Dragendorff, M: mayer, W: Wagner, B: Baljet, Fe: Ferric chloride, GS: Gelatin salt, K: Kedde, R: Raymond-Marthoud, KK: Keller-Kiliani, Sh: Shinoda, Ci: Citroboric, Es: Foam test, LB: Liebermann-Buchard, SW: Salkowski, VS: Vanillin-sulfuric acid, BO: Bornträger. Presence (+); absence (-).

Table S2. Phytocomponents identified in the MA-24F fraction of leaves of *M. alceifolia* Jacq., by GC-MS

No.	RT (min)	Molecular Formula	$[M]^+$	Compound	Type
1	14.297	$C_{15}H_{26}O_2$	238	Methyl 10,11-tetradecadienoate	Fatty acid derivative
2	15.418	$C_{15}H_{26}$	204	Santalane	sesquiterpenoids
3	19.946	$C_{18}H_{36}O$	268	2-Pentadecanone, 6,10,14-trimethyl	sesquiterpenoids
4	22.405	$C_{19}H_{34}O_2$	294	Methyl 9,10-octadecadienoate	Fatty acid derivative
5	22.559	$C_{20}H_{40}O$	296	Phytol	Diterpenoid
6	28.533	$C_{29}H_{50}O_4$	462	α-Tocospiro A	Tocopherol
7	28.682	$C_{29}H_{50}O_4$	462	α-Tocospiro B	Tocopherol
8	29.941	$C_{28}H_{48}O_2$	416	γ-Tocopherol	Tocopherol
9	30.273	$C_{31}H_{52}O_2$	456	β-Sitosterol acetate	Phytosterol
10	31.492	$C_{29}H_{48}O$	412	Stigmasterol	Phytosterol
11	32.001	$C_{29}H_{50}O$	414	(24R)-Stigmast-5-en-3-beta-ol	Phytosterol
12	32.390	$C_{30}H_{48}O_3$	456	Methyl commate A	Triterpene
13	32.842	$C_{30}H_{50}O$	426	Alpha Amyrin	Triterpene
14	33.317	$C_{45}H_{74}O$	631	Solanesol	Triterpene
15	33.620	$C_{30}H_{50}O$	426	epi-Swertenol	Triterpene

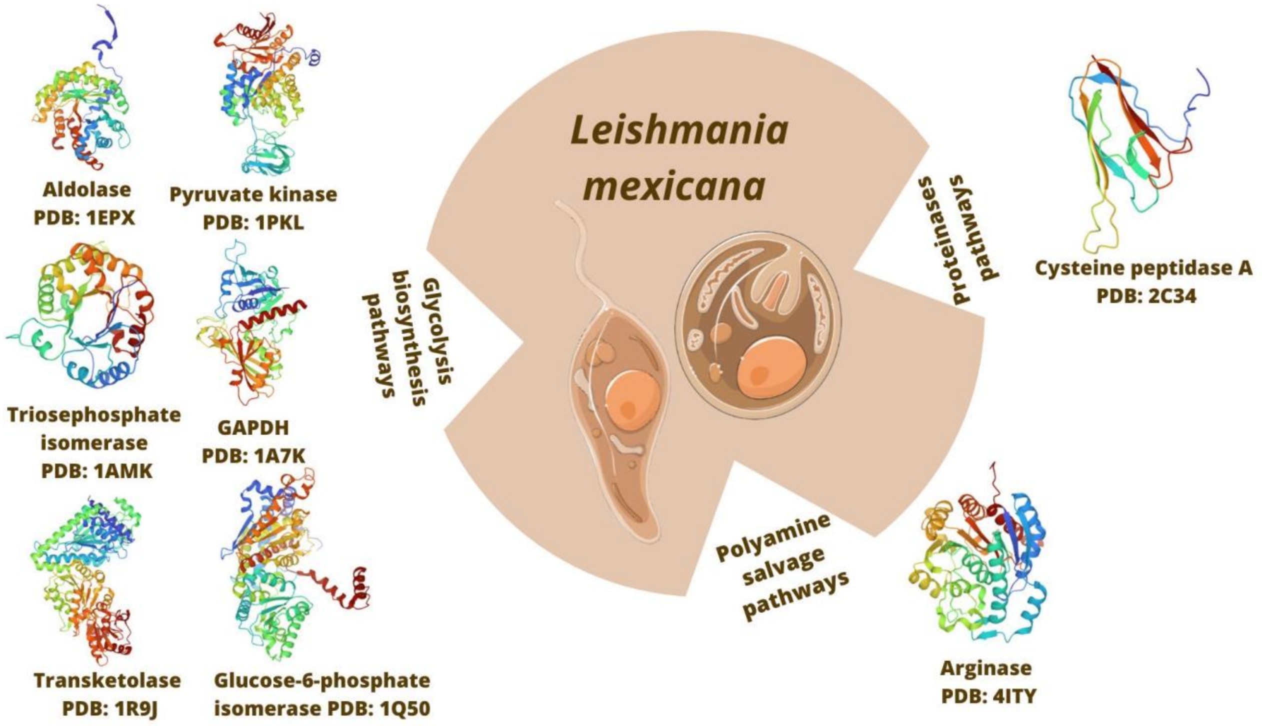

Figure S1. *Leishmania mexicana* protein targets. Protein Data Bank (PDB)

Table S3. Scoring Function estimation of binding affinity energy and molecular interactions of phytocomponents identified in the Ma-24F fraction of leaves of *M. alceifolia* by GC-MS upon docking in a potential allosteric sites of significant *L. mexicana* target proteins for AutoDock Vina program

Compounds	Allosteric GScore (Binding energy kcal/mol^{-1}) *							
	1PKL	1A7K	1AMK	1EPX	1Q50	19RJ	4ITY	2C34
Amphotericin B	-9.1 ± 0.1	-9.3± 0.1	-6.9± 0.2	-8.6 ± 0.1	-9.0± 0.1	-9.6± 0.1	-7.4± 0.2	-7.6± 0.2
Miltefosine	-4.9 ± 0.2	-4.5 ± 0.2	-4.3 ± 0.2	-5.4 ± 0.1	-5.1 ± 0.1	-5.0 ± 0.1	-4.9± 0.2	-4.4± 0.2
Methyl 10,11-tetradecadienoate	-5.0± 0.1	-3.5 ± 0.1	-4.3± 0.2	-4.1± 0.1	-4.1± 0.1	-4.3± 0.2	-4.0± 0.2	-4.0± 0.1
Santalane	-6.9± 0.2	-5.5± 0.2	-6.0± 0.1	-5.5± 0.1	-5.9± 0.2	-5.5± 0.2	-5.4± 0.2	-5.3± 0.1
2-Pentadecanone, 6,10,14-trimethyl	-4.5± 0.1	-4.0± 0.1	-4.2± 0.1	-4.1± 0.1	-4.2± 0.1	-4.5± 0.2	-4.0± 0.1	-4.3± 0.2
Methyl 9,10-octadecadienoate	-4.3± 0.1	-4.1± 0.1	-3.6± 0.1	-4.0± 0.1	-3.7± 0.1	-3.2± 0.1	-4.1± 0.1	-3.4± 0.1
Phytol	-6.2± 0.1	-5.1± 0.1	-5.0± 0.1	-5.0± 0.1	-5.4± 0.1	-4.8± 0.1	-4.7± 0.1	-4.8± 0.1
α-Tocospiro A	-7.6± 0.2	-6.3± 0.2	-5.6± 0.1	-7.0± 0.1	-5.8± 0.1	-6.5± 0.1	-5.8± 0.1	-6.1± 0.2
α-Tocospiro B	-7.3± 0.1	-6.3± 0.1	-6.8± 0.1	-6.7± 0.1	-5.9± 0.1	-6.6± 0.1	-6.6± 0.1	-5.8± 0.1
γ-Tocopherol	-6.2± 0.1	-6.4± 0.1	-6.1± 0.2	-6.6± 0.2	-6.2± 0.2	-6.5± 0.1	-6.5± 0.2	-5.9± 0.1
β-Sitosterol acetate	-6.8± 0.1	-7.9± 0.1	-6.4± 0.1	-7.0± 0.1	-6.7± 0.1	-7.1± 0.1	-6.7± 0.1	-6.9± 0.1
Stigmasterol	-7.8± 0.1	-7.5± 0.1	-7.1± 0.1	-7.2± 0.1	-7.0± 0.1	-7.1± 0.1	-7.2± 0.1	-7.4± 0.1
(24R)-Stigmast-5-en-3-beta-ol	-7.9± 0.1	-7.5± 0.1	-7.2± 0.2	-7.6± 0.2	-7.8± 0.1	-7.0± 0.1	-7.5± 0.1	-6.9± 0.1
Methyl commate A	-8.3 ± 0.2	-7.9± 0.1	-7.4± 0.1	-8.3± 0.1	-7.8± 0.2	-8.6± 0.1	-7.5± 0.2	-8.1± 0.1 (-6.0) **
α- Amyrin	-9.9 ± 0.1 (-9.1) **	-8.6 ± 0.1	-7.9± 0.2	-8.6± 0.1	-7.5± 0.2	-8.2 ± 0.1	-8.5 ± 0.1	-7.8± 0.2
Solanesol	-5.7± 0.1	-6.0± 0.1	-5.3± 0.2	-5.9± 0.2	-5.7± 0.1	-5.7± 0.1	-5.2± 0.2	-5.1± 0.1
Episwertenol	-8.5 ± 0.1	-9.1± 0.1	-8.3 ± 0.2	-8.2 ± 0.2	-7.6± 0.1	-8.2 ± 0.1	-8.7 ± 0.1 (-7.3) **	-8.0 ± 0.1

Glycolysis biosynthesis pathway proteins	Polyamine salvage pathway protein	Proteinase pathway protein
Pyruvate kinase (PDB: 1PKL), Glyceraldehyde-3-Phosphate Dehydrogenase (GAPDH) (PDB: 1A7K), triose phosphate isomerase (PDB: 1AMK), aldolase (PDB: 1EPX), phosphoglucose isomerase (PDB: 1Q50) transketolase (PDB: 1R9J)	Arginase (PDB: 4ITY)	Cysteine peptidase A (PDB: 2C34)

*Each binding energy (kcal/mol) represents the mean ± standard error of the mean of six independent replicas kcal/mol^{-1} n=6.

** Validated values using the CB-Dock program.

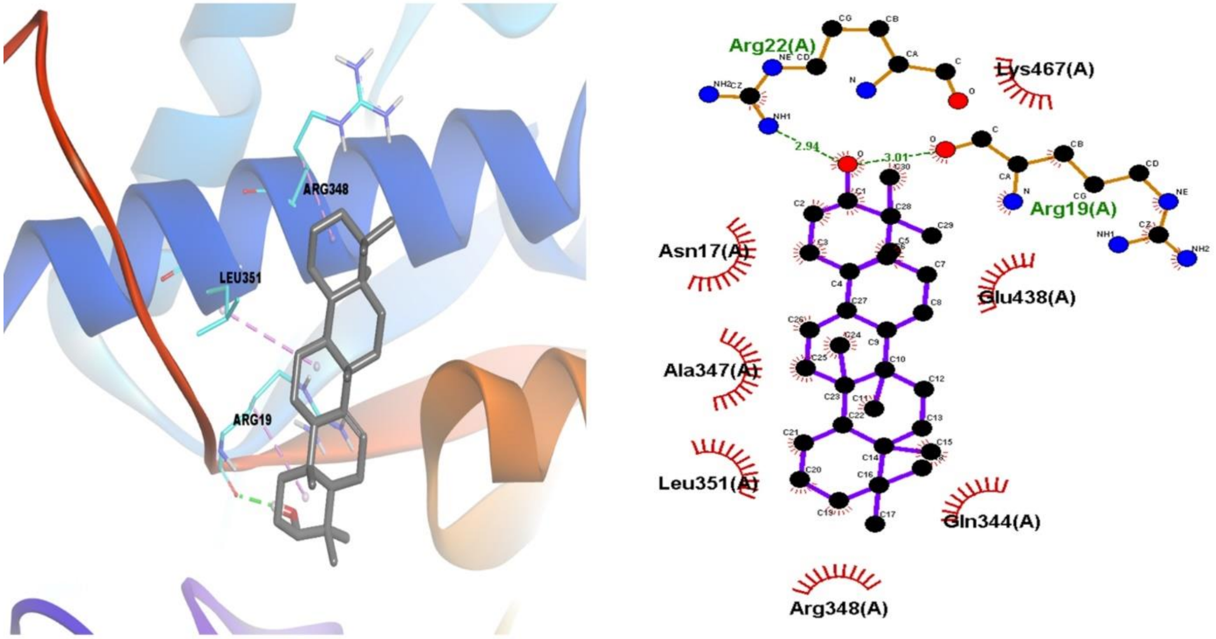

Figure S2a. Episwertenol compound interaction of allosteric binding sites of target proteins pyruvate kinase (PDB: 1PKL).

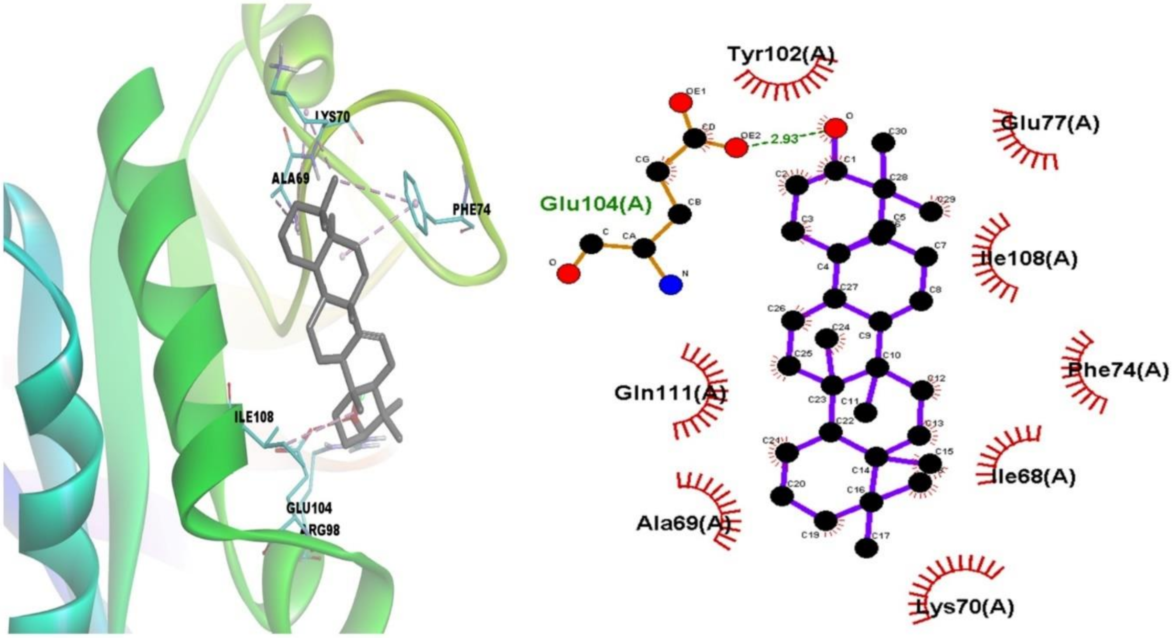

Figure S2b. Episwertenol compound interaction of allosteric binding sites of target triose phosphate isomerase (PDB: 1AMK).

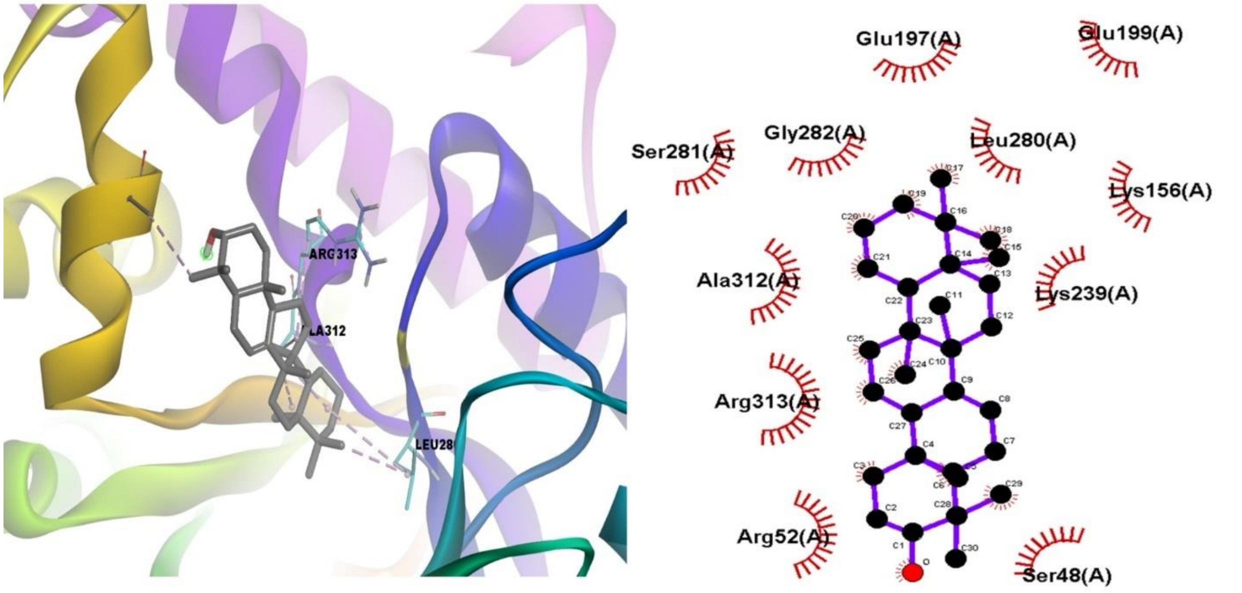

Figure S2c. Episwertenol compound interaction of allosteric binding sites of target aldolase (PDB: 1EPX).

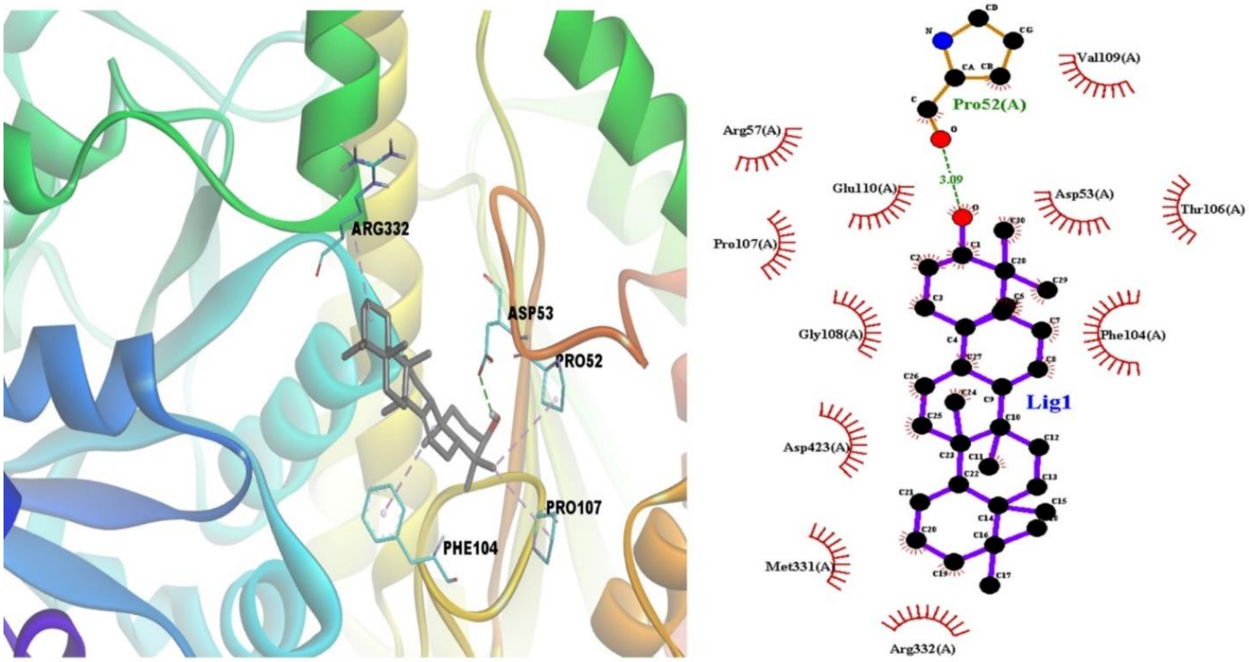

Figure S2d. Episwertenol compound interaction of allosteric binding sites of target transketolase (PDB: 1R9J).

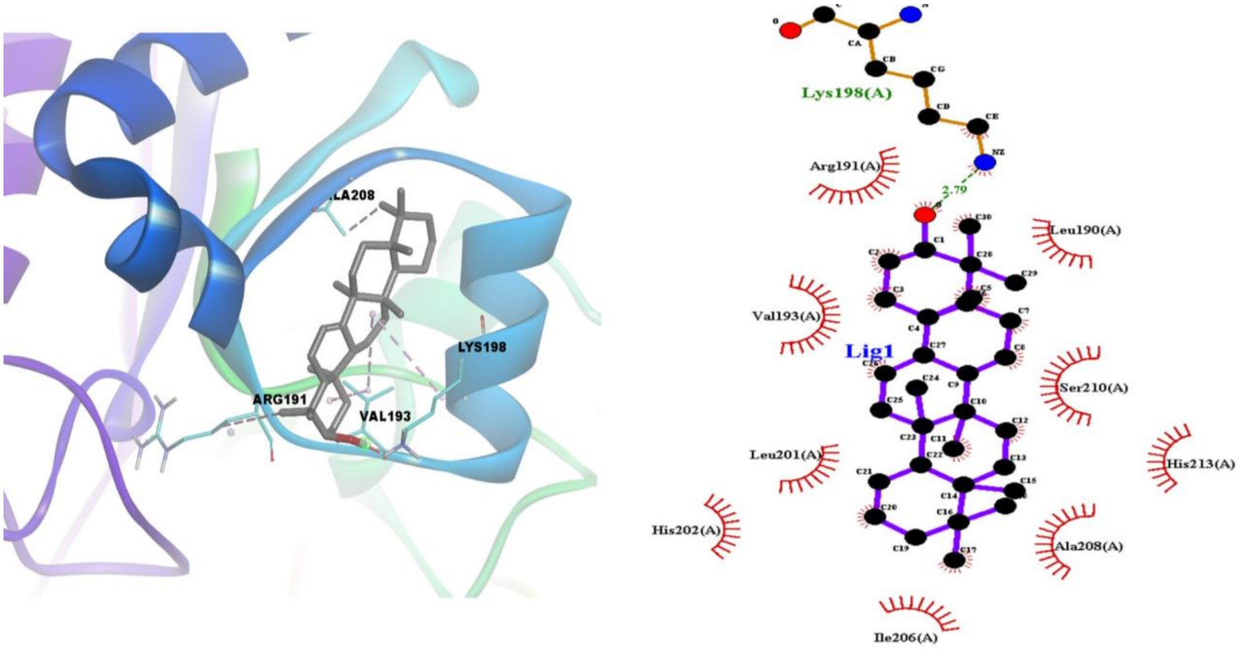

Figure S2e. Episwertenol compound interaction of allosteric binding sites of target arginase (PDB: 4ITY).

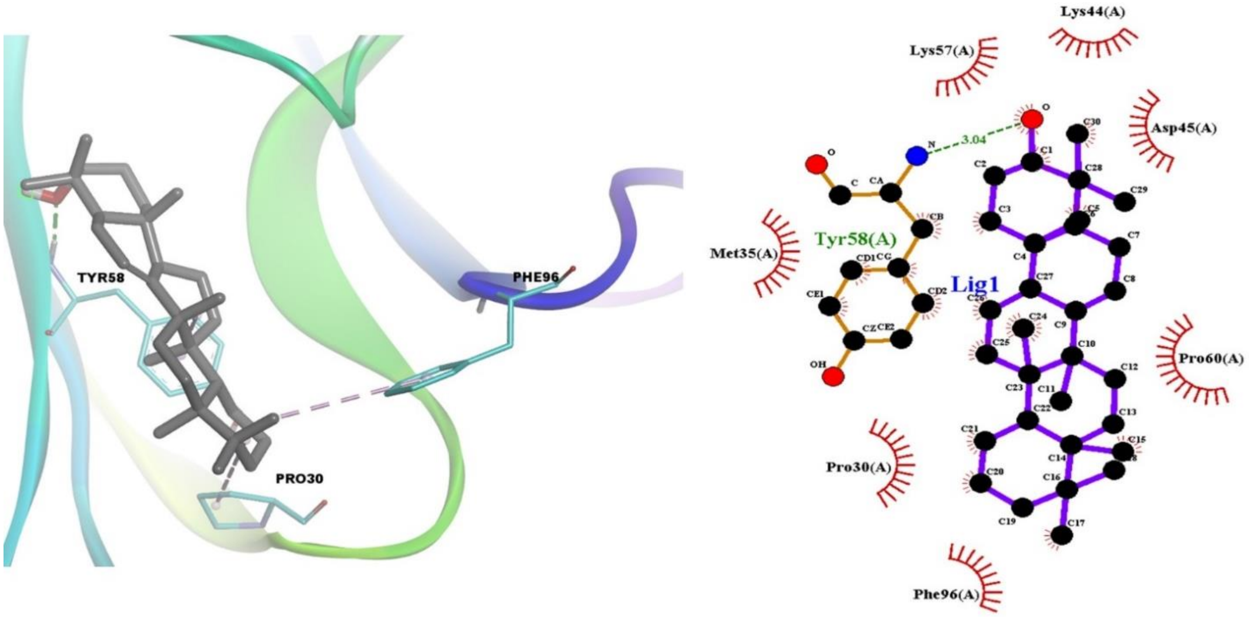

Figure S2f. Episwertenol compound interaction of allosteric binding sites of target Cysteine peptidases A (PDB: 2C34).

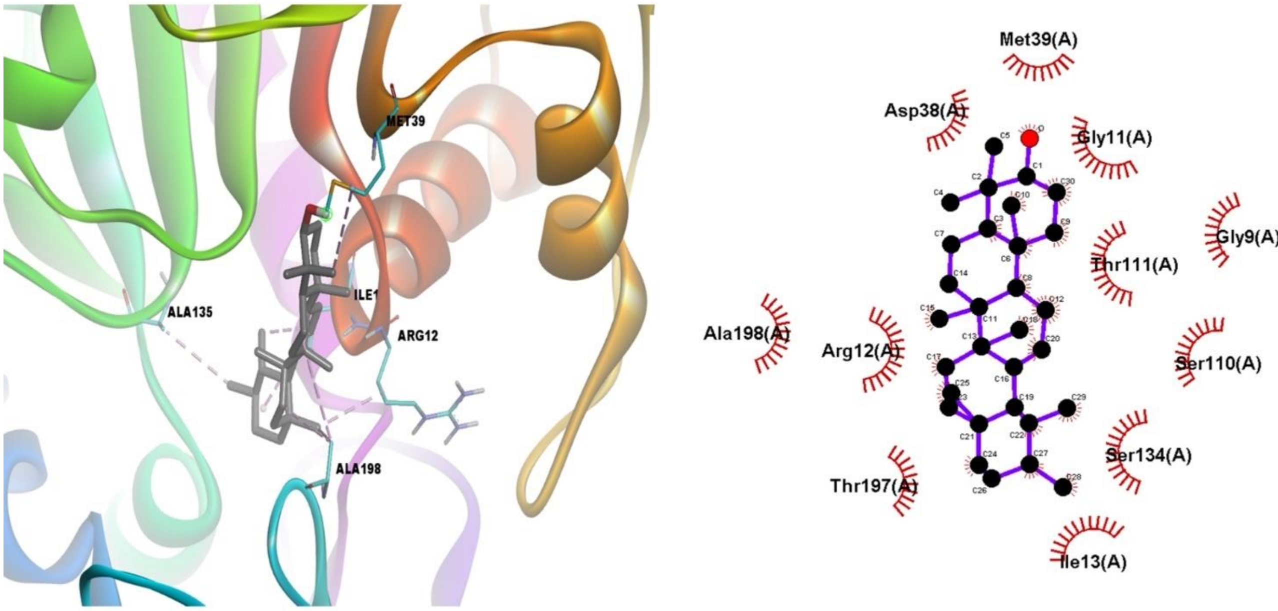

Figure S3a. α-Amiryn compound interaction of allosteric binding sites of target GAPDH (PDB: 1A7K).

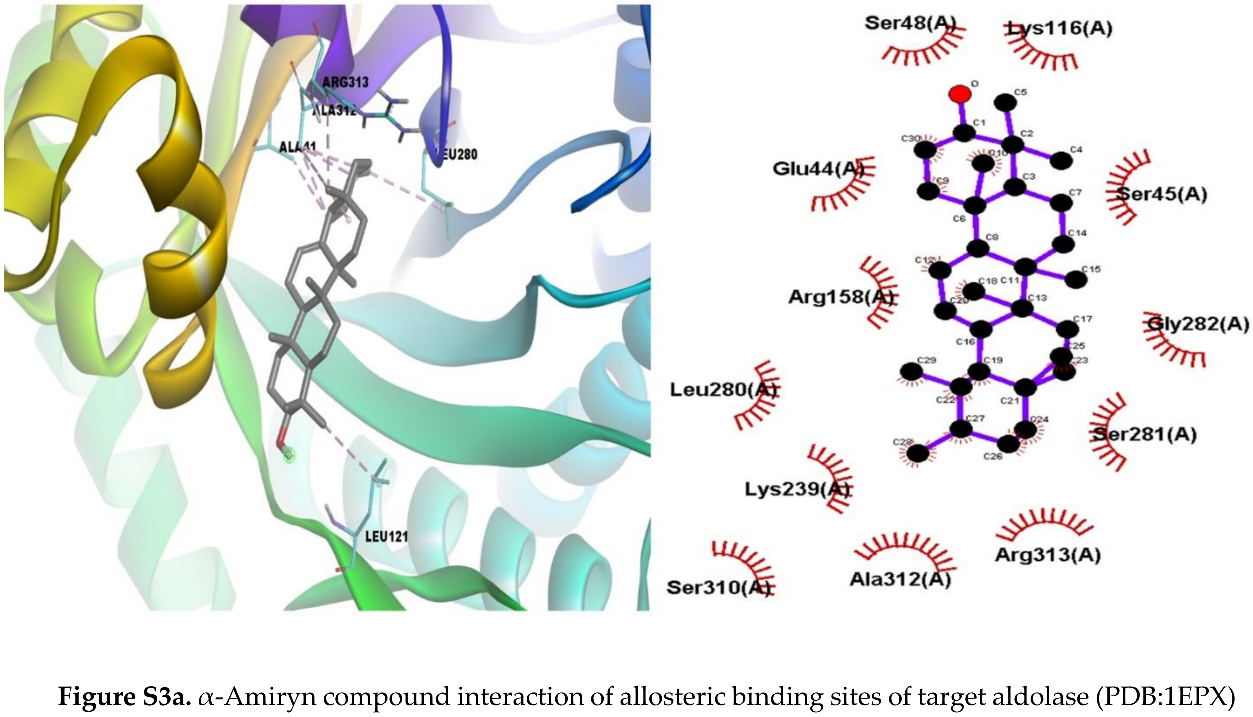

Figure S3a. α-Amiryn compound interaction of allosteric binding sites of target aldolase (PDB:1EPX)

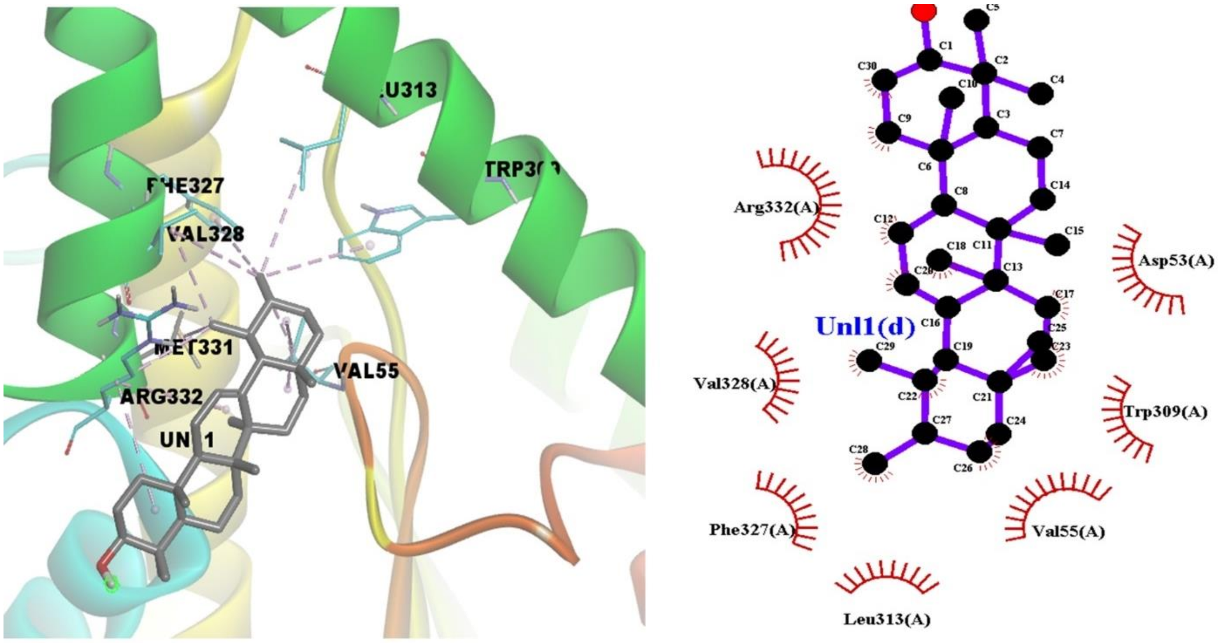

Figure S3b. α-Amiryn compound interaction of allosteric binding sites of target transketolase (PDB: 1R9J).

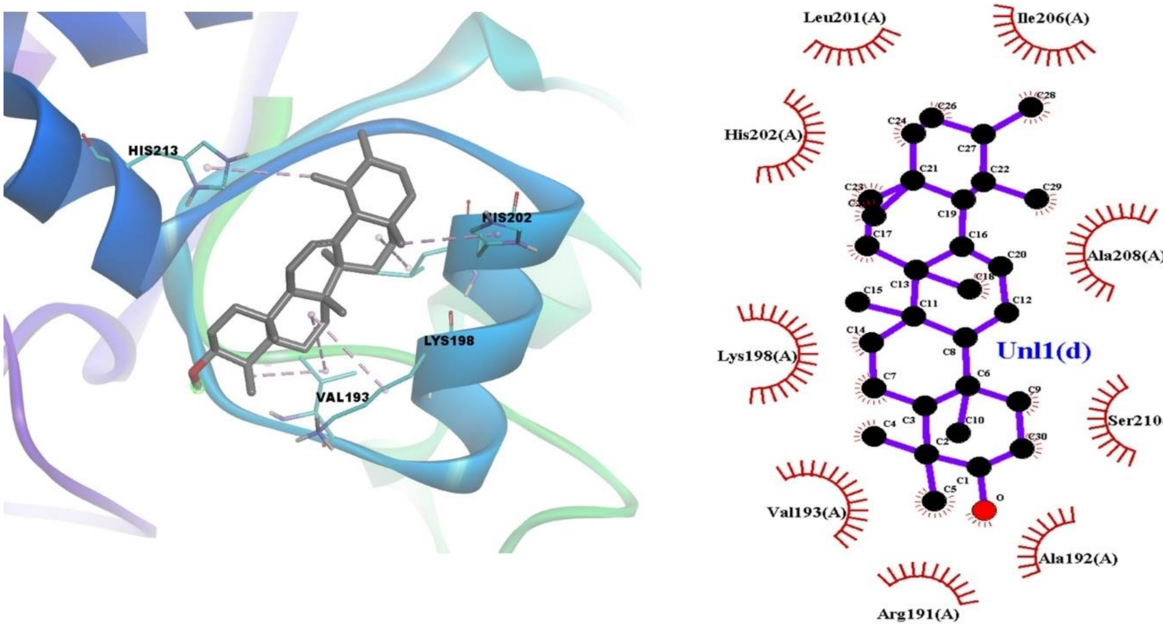

Figure S3c. α-Amiryn compound interaction of allosteric binding sites of target arginase (PDB: 4ITY).

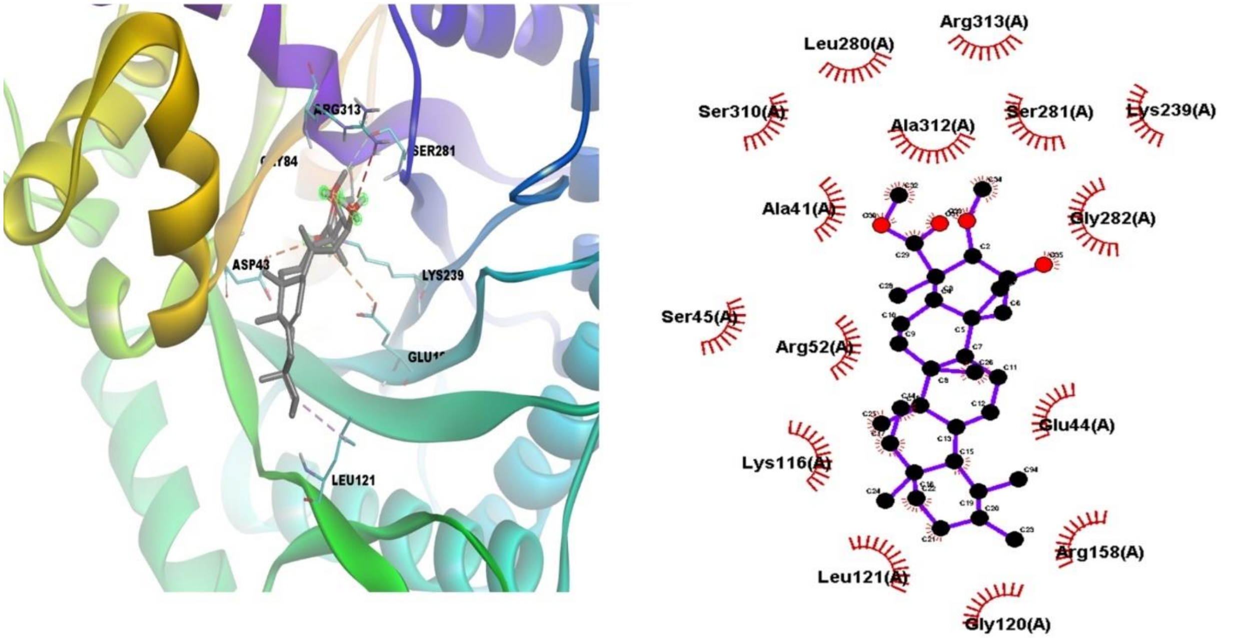

Figure S4a. Methyl commate A compound interaction of allosteric binding sites of target aldolase (PDB: 1EPX).

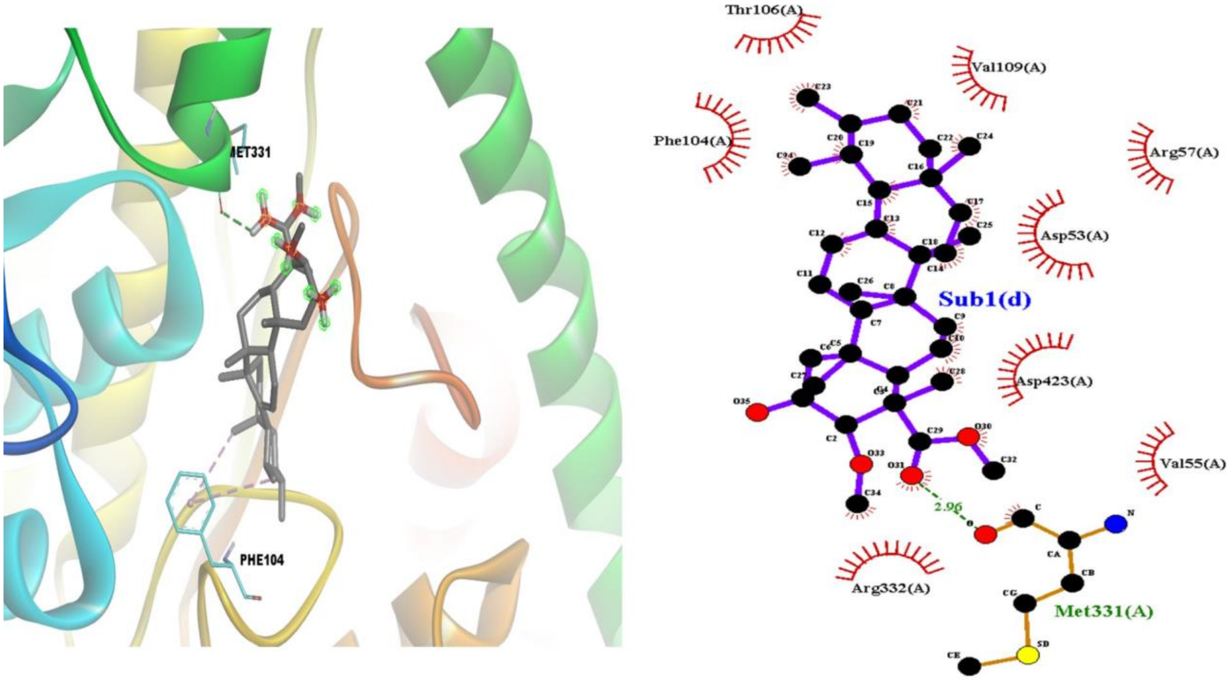

Figure S4b. Methyl commate A compound interaction of allosteric binding sites of target transketolase (PDB: 1R9J).

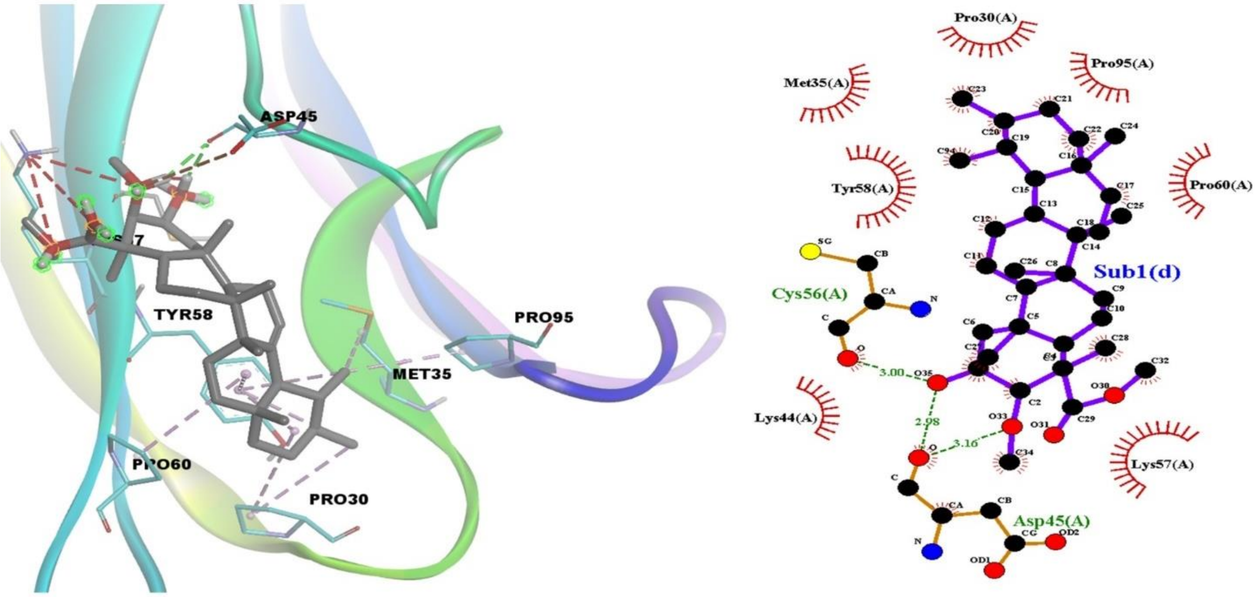

Figure S4c. Methyl commate A compound interaction of allosteric binding sites of target cysteine peptidases A (PDB: 2C34).

Table S4. Identification of amino acid residues in the allosteric site of proteins in *L. mexicana* selected for docking

Pathway	Proteins *L. mexicana*	Amino acid allosteric site residues	Size and shape descriptors	Surface descriptors
	GAPDH (PDB: 1A7K),	Arg12, Ile13, Gly11, Gly9, Phe10, Asp38, Met39, Thr111, Gly112, Leu113, Ser110, Ala135, Thr199, Ala198, Cys166, Thr197	Volume [Å^3]: 1166.66<br>Surface [Å^2]: 1497.66<br>Depth [Å]: 29.76	Apolar amino acid ratio 0.37<br>Polar amino acid ratio 0.47<br>Positive amino acid ratio 0.10<br>Negative amino acid ratio 0.07
Glycolysis biosynthesis pathway	Pyruvate kinase (PDB: 1PKL),	Gln40, Gly44, ILe41, Ser46, Leu74, Val76, Ile78, ASn77, Leu351, Cys428, ASn432, Phe463, Ser439, Glu438, Lys467	Volume [Å^3]: 1145.82<br>Surface [Å^2]:1320.34<br>Depth [Å]: 20.04	Apolar amino acid ratio 0.44<br>Polar amino acid ratio 0.37<br>Positive amino acid ratio 0.11<br>Negative amino acid ratio 0.07
	Triose phosphate isomerase (PDB: 1AMK)	Ala69, Lys70, Ile68, Phe74, Glu77, Arg98, Tyr102, Lys112, Ile108, Glu104, Gln111, Glu115	Volume [Å^3]: 951.62<br>Surface [Å^2]: 1297.45<br>Depth [Å]: 24.92	Apolar amino acid ratio 0.37<br>Polar amino acid ratio 0.43<br>Positive amino acid ratio0.09<br>Negative amino acid ratio 0.11
	Aldolase (PDB: 1EPX)	Ala41, Arg52, Glu44, Ser48, Ser45, Lys116, Gly120, Leu121, Lys156, Arg158, Leu280, Lys239, Ser281, Ser286, Gly282, Arg313, Ser310, Ala312, Tyr311	Volume [Å^3]: 1039.17<br>Surface [Å^2]: 1250.41<br>Depth [Å]: 25.51	Apolar amino acid ratio0.46<br>Polar amino acid ratio0.24<br>Positive amino acid ratio0.17<br>Negative amino acid ratio0.13
	Transketolase (PDB: 1R9J)	Pro52, Tpr54, Asp53, Leu33, Pro52, Arg57, Arg103, Phe104, Thr106, Pro107, Gly108, Glu110,Met331, Arg332, Phe327, Tpr309, Val55, Val328, Arg332, Asp423	Volume [Å^3]: 387.13<br>Surface [Å^2]: 316.33<br>Depth [Å]: 16.06	Apolar amino acid ratio 0.28<br>Polar amino acid ratio 0.50<br>Positive amino acid ratio 0.14<br>Negative amino acid ratio 0.06

**Polyamine salvage pathway**	Arginase (PDB: 4ITY)	Leu190, Arg19, Ala192, Val193, Lys198, Leu201, His202, Asn205, Ile206, Ala207, Ala208, Ser210, His212, His213, Val331, Arg332, Trp369	Volume [Å^3]: 548.16<br>Surface [Å^2]: 887.27<br>Depth [Å]: 16.94	Apolar amino acid ratio 0.58<br>Polar amino acid ratio 0.18<br>Positive amino acid ratio 0.12<br>Negative amino acid ratio 0.12
**Proteinase pathway**	Cysteine peptidase A (PDB: 2C34)	Thr31, Pro40, Met35, Gly43, Lys44, Asp45, Tyr58, Pro60, Val70, Phe96	Volume [Å^3] 489.86<br>Surface [Å^2] 1065.77<br>Depth [Å] 14.07	Apolar amino acid ratio 0.40<br>Polar amino acid ratio 0.25<br>Positive amino acid ratio 0.15<br>Negative amino acid ratio 0.20

Count. Table S4.

References

1. Cervantes-Ceballos, L.; Sánchez-Hoyos, J.; Sánchez-Hoyos, F.; Torres-Niño, E.; Mercado-Camargo, J.; Echeverry-Gómez, A.; Jotty Arroyo, K.; Del Olmo-Fernández, E.; Gómez-Estrada, H. An Overview of Genus *Malachra* L.-Ethnobotany, Phytochemistry, and Pharmacological Activity. *Plants* **2022**, *11*, 2808. https://doi.org/10.3390/plants11212808.
2. Gómez-Estrada, H.; Díaz-Castillo, F.; Franco-Ospina, L.; Mercado-Camargo, J.; Guzmán-Ledezma, J.; Medina, J.D.; Gaitán-Ibarra, R. Folk medicine in the northern coast of Colombia: An overview. *J. Ethnobiol. Ethnomed*. **2011**, *7*, 27. https://doi.org/10.1186/1746-4269-7-27.
3. Kvist, L.; Christensen, S.; Rasmussen, H.; Mejia, K.; Gonzalez, A. Identification and evaluation of Peruvian plants used to treat malaria and leishmaniasis. *J. Ethnopharmacol*. **2006**, *106*, 390–402. https://doi.org/10.1016/j.jep.2006.01.020.
4. López-Cuesta, B. Studio Ethnobotanics of Plants against Malaria. 2017. Available online: http://147.96.70.122/web/TFG/TFG/Memoria/Beatriz%20Cuesta%20Lopez.pdf (accessed on 18 November 2022).
5. World Health Organization. Leishmaniasis. 2021. Available online: https://www.who.int/data/gho/data/themes/topics/gho-ntd-leishmaniasis (accessed on 18 November 2022).
6. World Health Organization. Leishmaniasis. 2022. Available online: https://www.who.int/news-room/fact-sheets/detail/leishmaniasis (accessed on 18 November 2022).
7. Ruiz, L.; Ruiz, L.; Maco, M.; Cobos, M.; Gutierrez-Choquevilca, A.-L.; Roumy, V.Plants used by native Amazonian groups from the Nanay River (Peru) for the treatment of malaria. *J. Ethnopharmacol.* **2011**, *133*, 917–921. https://doi.org/10.1016/j.jep.2010.10.039.
8. Metzdorf, I.P.; Junior, M.S.; Matos, M.D.; de Souza Filho, A.F.; de Souza Tsujisaki, R.A.; Franco, K.G.; Shapiro, J.T.; de Almeida Borges, F. Molecular characterization of *Leishmania infantum* in domestic cats in a region of Brazil endemic for human and canine visceral leishmaniasis. *Acta Trop*. **2017**, *166*, 121–125. https://doi.org/10.1016/j.actatropica.2016.11.013.
9. Instituto Nacional de Salud. Boletín Epidemiológico Semanal. Semana Epidemiológica 25. 2022. Available online: https://www.ins.gov.co/buscador-eventos/BoletinEpidemiologico/2022_Bolet%C3%ADn_epidemiologico_semana_25.pdf (accessed on 18 November 2022).
10. Kaur, G.; Rajput, B. Comparative analysis of the omics technologies used to study antimonial, amphotericin B, and pentamidine resistance in leishmania. *J. Parasitol. Res*. **2014**, *2014*, 726328. https://doi.org/10.1155/2014/726328.
11. Silva-Almeida, M.; Pereira, B.A.; Ribeiro-Guimarães, M.L.; Alves, C.R. Proteinases as virulence factors in *Leishmania* spp. infection in mammals. *Parasit Vectors* **2012**, *7*, 160. https://doi.org/10.1186/1756-3305-5-160.

12. Silva-Almeida, M.; Souza-Silva, F.; Pereira, B.A.; Ribeiro-Guimaraes, M.L.; Alves, C.R. Overview of the organization of protease genes in the genome of *Leishmania* spp. *Parasit Vectors* **2014**, *7*, 387. https://doi.org/10.1186/1756-3305-7-387.
13. Chawla, B.; Madhubala, R. Drug targets in *Leishmania*. *J Parasit Dis*. **2010**, *34*, 1–13. https://doi.org/10.1007/s12639-010-0006-3.
14. Berman, H.; Westbrook, J.; Feng, Z.; Gilliland, G.; Bhat, T.N.; Weissig, H.; Shindyalov, I.N.; Bourne, P.E. The Protein Data Bank. *Nucleic Acids Res.* **2000**, *28*, 235–242. https://doi.org/10.1093/nar/28.1.235.
15. Padilla, C.A.; Alvarez, M.J.; Combariza, A.F. *Leishmania* Proteomics: An in Silico Perspective. *Preprints* **2019**, *4, 1-36*. doi: 10.20944/preprints201902.0122.v4.
16. Cervantes-Ceballos, L.; Sánchez-Hoyos, F.; Gómez-Estrada, H. Antibacterial activity of *Cordia dentata* Poir, *Heliotropium indicum* Linn and *Momordica charantia* Linn from the Northern Colombian Coast. *Rev. Colomb. Cienc. Químico-Farm.* **2017**, *42*, 143–159. https://doi.org/10.15446/rcciquifa.v46n2.67933.
17. Sen, R.; Chatterjee, M. Plant derived therapeutics for the treatment of Leishmaniasis. *Phytomedicine* **2011**, *18*, 1056–1069. https://doi.org/10.1016/j.phymed.2011.03.004.
18. Vargas, E.; Echeverri, F.; Upegui, Y.A.; Robledo, S.M.; Quiñones, W. Hydrazone Derivatives Enhance Antileishmanial Activity of Thiochroman-4-ones. *Molecules* **2017**, *23*, 70. https://doi.org/10.3390/molecules23010070.
19. Luque-Ortega, J.R.; Rivas, L. Characterization of the Leishmanicidal Activity of Antimicrobial Peptides. In *Antimicrobial Peptides: Methods in Molecular Biology*; Giuliani, A., Rinaldi, A., Eds.; Humana Press: Totowa, NJ, USA, 2010; Volume 618. https://doi.org/10.1007/978-1-60761-594-1_25.
20. Robledo, S.; Osorio, E.; Muñoz, D.; Jaramillo, L.M.; Restrepo, A.; Arango, G.; Vélez, I. In vitro and in vivo cytotoxicities and antileishmanial activities of thymol and hemisynthetic derivatives. *Antimicrob. Agents Chemother*. **2005**, *49*, 1652–1655. https://doi.org/10.1128/AAC.49.4.1652-1655.2005.
21. Coa, J.C.; García, E.; Carda, M.; Agut, R.; Vélez, I.D.; Muñoz, J.A.; Yepes, L.M.; Robledo, S.M.; Cardona, W.I. Synthesis, leishmanicidal, trypanocidal and cytotoxic activities of quinoline-chalcone and quinoline-chromone hybrids. *Med. Chem. Res.* **2017**, *26*, 1405–1414. https://doi.org/10.1007/s00044-017-1846-5.
22. Upegui-Zapata, Y.A.; Echeverri, F.; Quiñones, W.; Torres, F.; Nacher, M.; Rivas, L.I.; Meira, C.D.S.; Gedamu, L.; Escobar, G.; Archbold, R.; et al. Mode of action of a formulation containing hydrazones and saponins against leishmania spp. Role in mitochondria, proteases and reinfection process. *Int. J. Parasitol. Drugs Drug Resist*. **2020**, *13*, 94–106. https://doi.org/10.1016/j.ijpddr.2020.06.004.
23. Trott, O.; Olson, A.J. AutoDock Vina: Improving the speed and accuracy of docking with a new scoring function, efficient optimization, and multithreading. *J. Comput. Chem*. **2010**, *31*, 455–461. https://doi.org/10.1002/jcc.21334.
24. Mercado-Camargo, J.; Cervantes-Ceballos, L.; Vivas-Reyes, R.; Pedretti, A.; Serrano-García, M.L.; Gómez-Estrada, H. Homology modeling of leishmanolysin (gp63) from *Leishmania panamensis* and molecular docking of flavonoids. *ACS Omega* **2020**, *5*, 14741–14749. https://doi.org/10.1021/acsomega.0c01584.
25. BIOVIA. *Discovery Studio Modeling, Software Version*; Dassault Systèmes: San Diego, CA, USA, 2017.
26. Wallace, A.C.; Laskowski, R.A.; Thornton, J.M. LIGPLOT: A program to generate schematic diagrams of protein-ligand interactions. *Protein Eng.* **1995**, *8*, 127–134. https://doi.org/10.1093/protein/8.2.127.
27. Samal, D. In Silico Homology Modeling and Docking Study of Translationally Controlled Tumor Protein of Labeorohita. *Int. J. For. Fish. Res.* **2017**, *1*, 8–22. https://doi.org/10.22161/ijfaf.1.4.2.
28. Liu, Y.; Grimm, M.; Dai, W.T.; Hou, M.C.; Xiao, Z.X.; Cao, Y. CB-Dock: A web server for cavity detection-guided protein-ligand blind docking. *Acta Pharmacol. Sin*. **2020**, *41*, 138–144. https://doi.org/10.1038/s41401-19-0228-6.
29. Cao, Y.; Li, L. Improved protein-ligand binding affinity prediction by using a curvature-dependent surface-area model. *Bioinformatics* **2014**, *30*, 1674–1680. https://doi.org/10.1093/bioinformatics/btu104.
30. Volkamer, A.; Kuhn, D.; Rippmann, F.; Rarey, M. DoGSiteScorer: A web server for automatic binding site prediction, analysis and druggability assessment. *Bioinformatics* **2012**, *28*, 2074–2075. https://doi.org/10.1093/bioinformatics/bts310.
31. Michel, M.; Homan, E.J.; Wiita, E.; Pedersen, K.; Almlöf, I.; Gustavsson, A.L.; Lundbäck, T.; Helleday, T.; Warpman Berglund, U. In silico druggability assessment of the NUDIX hydrolase protein family as a workflow for target prioritization. *Front. Chem.* **2020**, *8*, 443. https://doi.org/10.3389/fchem.2020.00443.
32. Li, J.; Fu, A.; Zhang, L. An Overview of Scoring Functions Used for Protein–Ligand Interactions in Molecular Docking. *Interdiscip. Sci. Comput. Life Sci.* **2019**, *11*, 320–328. https://doi.org/10.1007/s12539-019-00327-w.

33. Almahmoud, S.; Zhong, H.A. Molecular Modeling of Allosteric Site of Isoform-Specific Inhibition of the Peroxisome Proliferator-Activated Receptor PPARγ. *Biomolecules* **2022**, *12*, 1614. https://doi.org/10.3390/biom12111614.
34. Laskowski, R.A.; Swindells, M.B. LigPlot+: Multiple Ligand À Protein Interaction Diagrams for Drug Discovery. *J. Chem. Inf. Model.* **2011**, *51*, 2778–2786. https://doi.org/10.1021/ci200227u.
35. Couillaud, J.; Leydet, L.; Duquesne, K.; Iacazio, G. The Terpene Mini-Path, a New Promising Alternative for Terpenoids Bio-Production. *Genes* **2021**, *12*, 1974. https://doi.org/10.3390/genes12121974.
36. Mahizan, N.A.; Yang, S.K.; Moo, C.L.; Song, A.A.; Chong, C.M.; Chong, C.W.; Abushelaibi, A.; Lim, S.E.; Lai, K.S. Terpene Derivatives as a Potential Agent against Antimicrobial Resistance (AMR) Pathogens. *Molecules* **2019**, *24*, 2631. https://doi.org/10.3390/molecules24142631.
37. Raimundo, V.D.; Carvalho, R.P.R.; Machado-Neves, M.; Marques-da-Silva, E.A. Effects of terpenes in the treatment of visceral leishmaniasis: A systematic review of preclinical evidence. *Pharmacol. Res*. **2022**, *177*, 106117. https://doi.org/10.1016/j.phrs.2022.106117.
38. Guerreo, T.; Vejarano, P.; Ochoa, R. Tamizaje fitoquímico y cuantificación de flavonoides totales de las hojas y flores de *Malachra alceifolia* Jacq. *Ethnobot. Res. Appl.* **2018**, *4*, 70–75.
39. Camargos, H.S.; Silva, A.H.M.; Anjos, J.L.V.; Alonso, A. Molecular dynamics and partitioning of di-tert-butyl nitroxide in stratum corneum membranes: Effect of terpenes. *Lipids* **2010**, *45*, 419–427. https://doi.org/10.1007/S11745-010-3407-2.
40. Youssefi, M.R.; Moghaddas, E.; Tabari, M.A.; Moghadamnia, A.A.; Hosseini, S.M.; Farash, B.R.; Ebrahimi, M.A.; Mousavi, N.N.; Fata, A.; Maggi, F.; et al. In vitro and in vivo effectiveness of carvacrol, thymol and linalool against *Leishmania infantum*. *Molecules* **2019**, *24*, 2072. https://doi.org/10.3390/molecules24112072.
41. Yasmin, H.; Adhikary, A.; Al-Ahdal, M.N.; Roy, S.; Kishore, U. Host–Pathogen Interaction in Leishmaniasis: Immune Response and Vaccination Strategies. *Immuno* **2022**, *2*, 218–254. https://doi.org/10.3390/immuno2010015.
42. Das, A.; Jawed, J.J.; Das, M.C.; Sandhu, P.; De, U.C.; Dinda, B.; Akhter, Y.; Bhattacharjee, S. Antileishmanial and immunomodulatory activities of lupeol, a triterpene compound isolated from Sterculia villosa. *Int. J. Antimicrob. Agents* **2017**, *50*, 512–522. https://doi.org/10.1016/j.ijantimicag.2017.04.022.
43. Bhattacharya, S.; Biswas, M.; Haldar, P.K. The triterpenoid fraction from Trichosanthes dioica root exhibits in vitro antileishmanial effect against *Leishmania donovani* promastigotes. *Pharmacogn. Res*. **2013**, *2*, 109–112. https://doi.org/10.4103/0974-8490.110540.
44. Cheuka, P.M.; Mayoka, G.; Mutai, P.; Chibale, K. The Role of Natural Products in Drug Discovery and Development against Neglected Tropical Diseases. *Molecules* **2016**, *22*, 58. https://doi.org/10.3390/molecules22010058.
45. Greve, H.L.; Kaiser, M.; Mäser, P.; Schmidt, T.J. Boswellic Acids Show In Vitro Activity against *Leishmania donovani*. *Molecules* **2021**, *26*, 3651. https://doi.org/10.3390/molecules26123651.
46. Montaser, M.M.; El-Sharnouby, M.E.; El-Noubi, G.; El-Shaer, H.M.; Khalil, A.A.; Hassanin, M.; Amer, S.A.; El-Araby, D.A. *Boswellia serrata* Resin Extract in Diets of Nile Tilapia, Oreochromis niloticus: Effects on the Growth, Health, Immune Response, and Disease Resistance to *Staphylococcus aureus*. *Animals* **2021**, *11*, 446. https://doi.org/10.3390/ani11020446.
47. Pramanik, P.; Chakraborti, S.; Bagchi, A.; Chakraborti, T. Bioassay-based *Corchorus capsularis* L. leaf-derived β-sitosterol exerts antileishmanial effects against *Leishmania* donovani by targeting trypanothione reductase. *Sci. Rep.* **2020**, *10*, 20440. https://doi.org/10.1038/s41598-020-77066-2.
48. de Souza, J.H.; Michelon, A.; Banhuk, F.W.; Staffen, I.V.; Klein, E.J.; da Silva, E.A.; Menolli, R.A. Leishmanicidal, Trypanocidal and Antioxidant Activity of Amyrin-Rich Extracts from *Eugenia pyriformis* Cambess. *Iran. J. Pharm. Res*. **2020**, *4*, 343–353. https://doi.org/10.22037/ijpr.2020.113368.14258.
49. Ioset, J.-R. Natural Products for Neglected Diseases: A Review. *Curr. Org. Chem.* **2008**, *8*, 643–666. https://doi.org/10.2174/138527208784577394.
50. Ali, R.; Tabrez, S.; Rahman, F.; Alouffi, A.S.; Alshehri, B.M.; Alshammari, F.A.; Alaidarous, M.A.; Banawas, S.; Dukhyil, A.A.B.; Rub, A. Antileishmanial Evaluation of Bark Methanolic Extract of *Acacia nilotica*: In Vitro and In Silico Studies. *ACS Omega* **2021**, *6*, 8548–8560. https://doi.org/10.1021/acsomega.1c00366.
51. Singh, N.; Mishra, B.B.; Bajpai, S.; Singh, R.K.; Tiwari, V.K. Natural product-based leads to fight against leishmaniasis. *Bioorg. Med. Chem*. **2014**, *22*, 18–45. https://doi.org/10.1016/j.bmc.2013.11.048.
52. McKerrow, J.H.; Caffrey, C.; Kelly, B.; Loke, P.; Sajid, M. Proteases in parasitic diseases. *Annu. Rev. Pathol*. **2006**, *1*, 497–536. https://doi.org/10.1146/annurev.pathol.1.110304.100151.
53. Elmahallawy, E.K.; Alkhaldi, A.A.M. Insights into *Leishmania* Molecules and Their Potential Contribution to the Virulence of the Parasite. *Vet. Sci*. **2021**, *8*, 33. https://doi.org/10.3390/vetsci8020033.

54. Cordeiro, A.T.; Michels, P.A.; Delboni, L.F.; Thiemann, O.H. The crystal structure of glucose-6-phosphate isomerase from *Leishmania mexicana* reveals novel active site features. *Eur. J. Biochem*. **2004**, *271*, 2765–2772. https://doi.org/10.1111/j.1432-1033.2004.04205.x.
55. Smith, B.O.; Picken, N.C.; Westrop, G.D.; Bromek, K.; Mottram, J.C.; Coombs, G.H. The structure of *Leishmania mexicana* ICP provides evidence for convergent evolution of cysteine peptidase inhibitors. *J. Biol. Chem*. **2006**, *281*, 5821–5828. https://doi.org/10.1074/jbc.M510868200.
56. D'Antonio, E.L.; Ullman, B.; Roberts, S.C.; Dixit, U.G.; Wilson, M.E.; Hai, Y.; Christianson, D.W. Crystal structure of arginase from *Leishmania mexicana* and implications for the inhibition of polyamine biosynthesis in parasitic infections. *Arch. Biochem. Biophys*. **2013**, *535*, 163–176. https://doi.org/10.1016/j.abb.2013.03.015.
57. Jain, S.; Sahu, U.; Kumar, A.; Khare, P. Metabolic Pathways of *Leishmania* Parasite: Source of Pertinent Drug Targets and Potent Drug Candidates. *Pharmaceutics* **2022**, *14*, 1590. https://doi.org/10.3390/pharmaceutics14081590.
58. Rajasekaran, R.; Chen, Y.P. Potential therapeutic targets and the role of technology in developing novel antileishmanial drugs. *Drug Discov. Today* **2015**, *20*, 958–968. https://doi.org/10.1016/j.drudis.2015.04.006.
59. Kapil, S.; Singh, P.K.; Silakari, O. An update on small molecule strategies targeting leishmaniasis. *Eur. J. Med. Chem*. **2018**, *157*, 339–367. https://doi.org/10.1016/j.ejmech.2018.08.012.
60. Dorlo, T.P.; Balasegaram, M.; Beijnen, J.H.; de Vries, P.J. Miltefosine: A review of its pharmacology and therapeutic efficacy in the treatment of leishmaniasis. *J. Antimicrob. Chemother.* **2012**, *67*, 2576–2597. https://doi.org/10.1093/jac/dks275.
61. de Morais, S.M.; Vila-Nova, N.S.; Bevilaqua, C.M.; Rondon, F.C.; Lobo, C.H.; Moura, A.D.; Sales, A.D.; Rodrigues, A.P.; de Figuereido, J.R.; Campello, C.C.; et al. Thymol and eugenol derivatives as potential antileishmanial agents. *Bioorg. Med. Chem*. **2014**, *22*, 6250–6255. https://doi.org/10.1016/j.bmc.2014.08.020.
